# Phase Transformation and Electrochemical Behavior of Hexagonal TiO_2_ Nanotubes Under Different Annealing Temperatures and Heating Rates

**DOI:** 10.3390/mi17060757

**Published:** 2026-06-22

**Authors:** Aleksandra Jędrzejewska, Katarzyna Arkusz

**Affiliations:** Institute of Materials and Biomedical Engineering, Faculty of Engineering and Technical Sciences, University of Zielona Gora, 9 Licealna Street, 65-417 Zielona Gora, Poland; a.jedrzejewska@iimb.uz.zgora.pl

**Keywords:** hexagonal titanium dioxide nanotubes, biomedical, corrosion resistance, phase composition, thermal annealing, heating/cooling rate

## Abstract

In this study, hexagonal titanium dioxide nanotubes (hTNTs) fabricated by sonoelectrochemical anodization were thermally modified in air to investigate the influence of annealing temperature and heating/cooling rate on phase evolution, structural stability and electrochemical behavior. The samples were annealed at 450 °C, 550 °C, and 650 °C for 2 h using heating/cooling rates of 6 °C/min, 10 °C/min, and 20 °C/min. The hexagonal nanotubular morphology remained preserved after thermal treatment. However, increasing annealing temperature and heating/cooling rate promoted crack formation due to the thermally induced stress relaxation and phase transformation. The anatase content increased with increasing heating/cooling rate, indicating kinetically limited anatase-to-rutile transformation, whereas annealing at 650 °C promoted partial rutile formation. Electrochemical studies demonstrated that annealing temperature and heating/cooling rate affected the electrochemical behavior of hTNTs through different mechanisms. Increasing annealing temperature promoted structural ordering and partial anatase-to-rutile transformation, leading to reduced current response and enhanced electrochemical stability. In contrast, heating/cooling rate significantly affected impedance behavior and diffusion-related processes, indicating changes in charge transfer kinetics and ion transport within the nanotubular oxide layer. The results demonstrate that thermal treatment kinetics play an important role in controlling the phase composition and electrochemical behavior of hTNTs, providing insight into the thermal optimization of hexagonal TiO_2_ nanotubes for advanced functional applications.

## 1. Introduction

Titanium and its nanostructured derivatives are increasingly used in advanced technological applications, including biosensors, chemical processing equipment, aerospace components and biomedical implants [1,2,3]. Among them, titanium dioxide nanotubes have attracted considerable attention because of their high surface-to-volume ratio, chemical stability and favorable electrochemical and biological properties [4,5,6]. Conventional circular TiO_2_ nanotubes (TNTs) exhibit excellent biocompatibility and functional versality; however, their practical application may be limited by insufficient mechanical stability, irregular packing geometry, and relatively weak adhesion to the titanium substrate [7,8]. These limitations are particularly important in long-term electrochemical and biomedical application, where structural integrity and interfacial stability are crucial.

To overcome these limitations, hexagonal titanium dioxide nanotubes (hTNTs) have emerged as a structurally improved alternative to conventional circular nanotubes. The morphology and structural organization of hTNTs strongly depend on the fabrication strategy and anodization conditions. Hexagonal TiO_2_ nanotubes have been successfully fabricated using one-step anodization [9], multi-step (two- and three-step) anodization [10], pulse anodization [11], sonoelectrochemical anodization [12], anodization in sol-based electrolytes [13], and template-assisted growth via atomic layer deposition [14]. In most cases, the final morphology is governed by anodization parameters such as applied potential, anodization time, electrolyte composition, fluoride ion concentration, and the presence of complexing agents [11,13,15,16,17,18]. Depending on the processing conditions, hTNTs with diagonals from 33 nm to 230 nm and heights from 0.1 µm to 41.1 µm can be obtained.

Such geometric variability significantly affects the physicochemical and functional properties of hTNTs. Previous studies demonstrated that increasing anodization voltage from 10 V to 80 V systematically increased the nanotube diagonal from ~30 nm to ~200 nm and the nanotube height from ~1 µm to ~31 µm [12]. These morphology-dependent changes directly influence porosity, specific surface area, charge transport pathways, corrosion resistance, and mechanical response [12,19]. Electrochemical investigations revealed that hTNTs exhibit lower impedance and higher conductivity than compact TiO_2_ layers, while larger nanotube diagonals promote enhanced charge transfer accompanied by increased electrolyte accessibility and reduced corrosion resistance due to fluoride-containing defect incorporation during anodization [19]. Simultaneously, nanoindentation studies demonstrated that increasing nanotube dimensions reduces hardness and Young’s modulus, although hTNTs still exhibit superior mechanical performance compared with conventional circular TNTs, with reported Young’s modulus values in the range of 54–99 GPa [12], exceeding those of circular TNTs (4–43 GPa [20,21,22,23]).

As-fabricated TiO_2_ nanotubes, including hTNTs, are predominantly amorphous and therefore require post-anodization thermal treatment to induce crystallization into anatase and rutile phases [24,25]. In conventional TNT systems, annealing within the temperature range of approximately 300–600 °C promotes structural ordering and crystallization while preserving the nanotubular morphology [26,27,28]. The initial nucleation of anatase typically occurs between 300 °C and 450 °C [26], whereas further temperature increase progressively promotes the anatase-to-rutile transformation accompanied by grain growth and lattice reorganization [28]. Temperatures exceeding ~650 °C may lead to structural degradation and partial collapse of the nanotube architecture [28]. Excessive thermal exposure may lead to sintering effects, wall thickening, structural destabilization, and partial collapse of the nanotubular framework [28]. The transition from amorphous to crystalline TiO_2_ significantly alters the physicochemical characteristics of nanotube layers, including charge transport properties, corrosion resistance, dielectric behavior, mechanical stability, and interfacial electrochemical response [29,30].

Beyond the annealing temperature, the kinetics of thermal treatment may play an important role in determining the functional properties of hTNTs. Heating and cooling rates are controlling factors of crystallization, phase composition, and functional properties of TiO_2_ [25,31]. Galizia et al. [32] observed that higher heating and cooling rates promote a more pronounced transformation from anatase to rutile, highlighting the kinetic sensitivity of phase transitions. Conversely, Sierra-Uribe et al. [33] found that lower heating rates promoted the formation of highly crystalline anatase with preferential orientation in TiO_2_ nanotubes, which was associated with improved photoelectrocatalytic activity. Overall, these studies indicated that thermal kinetics is an important factor influencing microstructural evolution and functional properties in TiO_2_ systems. However, the effect of heating/cooling rates has not yet been systematically examined for hTNTs.

Despite extensive investigations of thermal treatment effects in conventional circular TNTs, the thermal behavior of hTNTs remains insufficiently explored. Due to their distinct hexagonal geometry, modified stress distribution, and different wall organization, hTNTs may exhibit fundamentally different phase transformation pathways, heat transfer, stress relaxation, defect redistribution and thermal stability compared with circular nanotubes. These structural modifications are expected to directly affect electrochemical performance by modifying charge transport pathways, interfacial charge transfer resistance, and ion accessibility within the nanotubular architecture. In particular, the influence of heating and cooling kinetics on crystallization dynamics, defect redistribution, diffusion processes, and electrochemical charge transfer mechanisms has not yet been systematically investigated. Understanding these relationships is fundamentally important because thermal kinetics may provide an additional processing parameter for tuning properties without altering nanotube geometry or chemical composition.

Therefore, this study investigates the influence of annealing temperature and heating/cooling rate on the phase composition, crystallinity, morphology, and electrochemical behavior of hexagonal TiO_2_ nanotubes fabricated by sonoelectrochemical anodization. Particular attention is devoted to understanding the relationships between thermal kinetics, phase evolution, structural reorganization, and electrochemical response, providing insight into the rational optimization of hTNTs for advanced electrochemical and biomedical applications.

## 2. Materials and Methods

### 2.1. Materials

All chemicals and reagents were purchased from Sigma-Aldrich (St. Louis, MO, USA) and used without further purification. Hexagonal TiO_2_ nanotubes (hTNTs) were fabricated on titanium foil (99.7% purity, 0.25 mm thickness). Ethylene glycol (≥99.8%), ammonium fluoride (NH_4_F, ≥98%), and disodium dihydrogen ethylenediaminetetraacetate (Na_2_[H_2_EDTA], ≥99.6%) were used for anodization process. Ringer’s solution consisting of 1.125 g of NaCl, 0.0525 g of KCl, 0.03 g CaCl_2_ and 0.025 g NaHCO_3_ was used as the electrolyte for all electrochemical measurements conducted at room temperature.

### 2.2. Formation of Hexagonal TiO_2_ Nanotubes

Prior to anodization, titanium foil was ultrasonically cleaned in acetone and distilled water, followed by drying under nitrogen stream. Hexagonal TiO_2_ nanotubes were fabricated by sonoelectrochemical anodization using a two-electrode configuration, in which platinum grid served as the counter electrode and titanium foil as the working electrode. The anodization process was carried out in an ethylene glycol-based electrolyte (94%) containing 0.3 wt.% NH_4_F and 0.1 wt.% Na_2_[H_2_EDTA]. The apparent pH of the electrolyte was adjusted to 6.1 at 25 °C (measured using a standard glass electrode; aqueous calibration buffers). Sonoelectrochemical anodization was performed using an Autolab PGSTAT302N potentiostat (Metrohm, Herisau, Switzerland) combined with ultrasound irradiation generated by a USC–T ultrasonic cleaner (VWR, Darmstadt, Germany) operating at 45 kHz and 200 W. During anodization, a constant potential of 20 V was maintained for 1 h.

The morphology and elemental composition of the hTNTs were characterized by field emission scanning electron microscopy (FE-SEM, JEOL JSM-7600F, Akishima, Tokyo, Japan) coupled with energy-dispersive X-ray spectroscopy (EDS, Oxford Instruments, Abingdon, United Kingdom). The morphological parameters of hTNTs, including diagonal and height, were determined from SEM micrographs using PC-SEM software ver 1.0.2.4. A total of 100 measurements were obtained from three randomly selected regions of each sample, which were also used for EDS mapping.

### 2.3. Thermal Modification of Hexagonal TiO_2_ Nanotubes

The hTNT samples were annealed in air using a PRC 265X150/100Z furnace (CZYLOK, Jastrzębie-Zdrój, Poland). To evaluate the influence of heating/cooling rate, the samples were annealed at 550 °C for 2 h using thermal rates of 6 °C/min, 10 °C/min, and 20 °C/min. The effect of annealing temperature was investigated by thermal treatment at 450 °C, 550 °C, and 650 °C for 2 h using a constant heating/cooling rate of 10 °C/min.

Raman spectroscopy was used to investigate the phase composition and structural changes induced by thermal treatment using inVia Raman spectrometer (Renishaw, Warszawa, Poland) with laser excitation at 532 nm (Ar^+^ laser, 1800 lines/mm grating, visible range). Raman spectra were collected using 1% of laser power, an exposure time of 10 s, and 5 accumulations. The anatase phase was identified based on characteristic Raman bonds located at 140 cm^−1^ (Eg), 398 cm^−1^ (B1g), 525 cm^−1^ (Eg), and 640 cm^−1^ (Eg). The anatase phase content was determined according to the methodology described in our previous studies [34,35].

### 2.4. Electrochemical Measurements

Open-circuit potential (OCP), cyclic voltammetry (CV) and electrochemical impedance spectroscopy (EIS) measurements were performed in a three-electrode configuration was using hTNTs as the working electrode. Electrochemical measurements were carried out using an Autolab PGSTAT302N (Metrohm, Herisau, Switzerland), with a Ag/AgCl reference electrode (E_Ag/AgCl_ = 222 mV vs. standard hydrogen electrode) and a platinum grid as counter electrode. The OCP tests were carried out for 1800 s prior to EIS measurements. Subsequently, EIS spectra were recorded immediately after OCP stabilization at the OCP value, over the frequency range from 100 kHz to 100 mHz, using a sinusoidal signal amplitude of 10 mV. Cyclic voltammetry measurements were performed within the potential range from −1 V to 1 V at a scan rate of 50 mV/s. The electrochemical double-layer capacitance (Cdl) was determined from cyclic voltammetry measurements performed in a non-faradaic potential window ranging from 100 to 300 mV. The measurements were carried out at scan rates of 10, 20, 40, 50, and 80 mV/s in Ringer’s solution. The capacitive current density was determined at 200 mV and plotted as a function of scan rate. The double-layer capacitance was calculated from the slope of the linear fit of the current density versus scan rate relationship. Statistical analysis of the capacitance values derived from cyclic voltammetry measurements was performed using one-way analysis of variance (ANOVA) to assess overall differences among the investigated groups. Subsequently, pairwise comparisons were carried out between the unannealed hTNT sample, used as a reference group, and each annealed sample using a two-tailed Welch’s *t*-test (*n* = 3). Welch’s *t*-test was selected because it does not assume equal variances between groups and is considered more robust for small sample sizes. Differences were considered statistically significant at *p* < 0.01.

All data were presented as mean ± standard deviation (SD) [12].

## 3. Results and Discussion

### 3.1. Influence of Heating/Cooling Rate on Phase Composition and Electrochemical Properties of hTNTs

**Figure 1:**
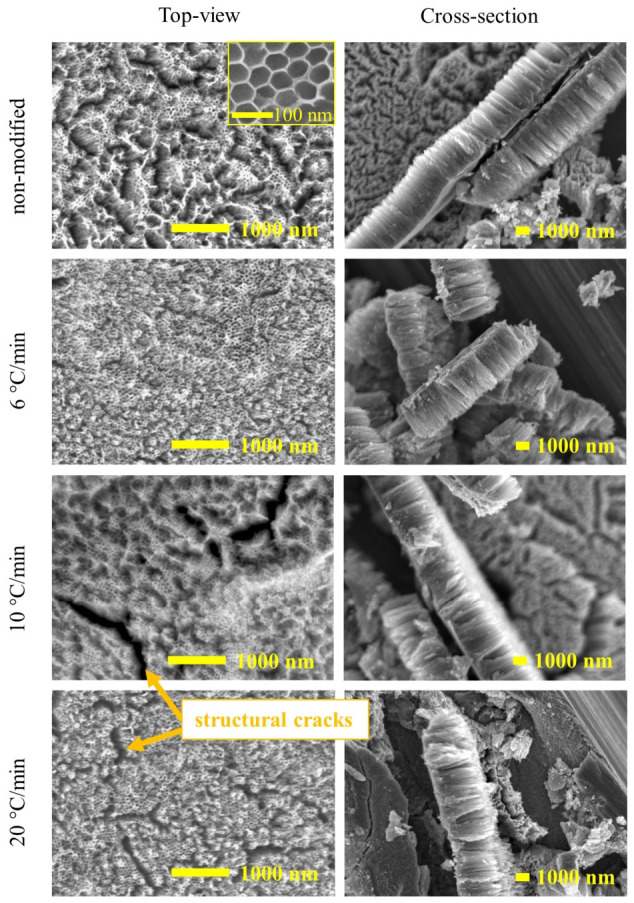
SEM images of hexagonal titanium dioxide nanotubes (hTNTs) thermally modified in air at 550 °C for 2 h using heating/cooling rates of 6 °C/min, 10 °C/min, and 20 °C/min.

Scanning electron microscopy analysis, as shown in Figure 1, confirmed the distinct hexagonal morphology of hTNTs synthesized via sonoelectrochemical anodization, a process previously developed and optimized in our previous research [12]. The fabricated hTNTs exhibited an average diagonal of 43 ± 4 nm and a height of 3755 ± 319 nm (Table 1). Thermal treatment of hTNTs at 550 °C in air for 2 h using different heating/cooling rates did not significantly affect the diagonal or height of the hexagonal titanium dioxide nanotubes (Table 1), indicating good thermal stability of the hTNT architecture. Raza et al. confirmed that the thermal ramping rate did not significantly affect the morphology of TiO_2_ nanotubes [25]. However, distinct differences in crack morphology were observed after annealing. The lowest heating/cooling rate (6 °C/min) resulted in a reduced number of cracks in the hTNT layer, whereas increasing heating/cooling rates promoted the formation of wider cracks extending deeper into oxide layer. These observations suggest that heating/cooling kinetics influence stress evolution and relaxation processes occurring during crystallization of the amorphous TiO_2_ layer. Faster heating/cooling rates may promote localized stress accumulation associated with rapid structural reorganization and phase evolution, leading to intensified crack propagation within the hTNT layer. Such crack development may additionally affect the electrochemical response by modifying electrolyte penetration pathways and the electrochemically active surface area.

The energy-dispersive X-ray spectroscopy (EDS) results presented in Table 1 confirmed the presence of fluorine in non-annealed hTNTs. The detected fluorine originates from the fluoride-containing electrolyte used for anodization, consistent with previous observations [19,36,37]. After annealing in air, fluorine was removed from hTNTs, indicating thermally induced removal of fluoride-containing species from the oxide layer [38,39,40,41].

Only minor variations in the Ti/O ratio were observed among samples annealed using different heating/cooling rates, suggesting that thermal treatment did not significantly affect the overall oxide stoichiometry. Nevertheless, differences in thermal kinetics may influence the defect structure and local distribution of oxygen-related defects within the TiO_2_ layer [42,43]. Together with the observed crack evolution, these changes may contribute to variations in electrochemical behavior by modifications in charge transfer, electrolyte penetration pathways, and the electrochemically active surface area of hTNTs.

**Figure 2:**
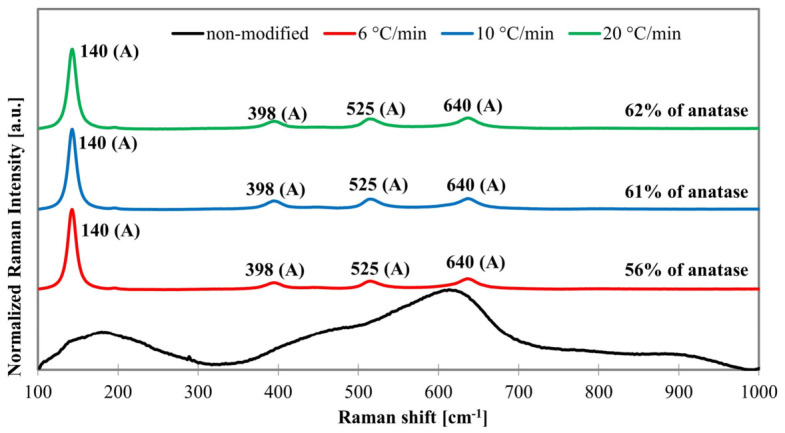
Raman spectra of hTNTs before and after thermal modification in air at 550 °C for 2 h using different heating/cooling rates: 6 °C/min, 10 °C/min, and 20 °C/min.

The Raman spectra presented in Figure 2 reveal clear differences between non-annealed and annealed hTNTs. Non-annealed samples exhibited weak and poorly defined Raman bands, confirming the predominantly amorphous character of the as-formed oxide layer [24]. In contrast, annealed hTNTs displayed well-defined Raman peaks corresponding to the anatase phase of TiO_2_. The dominant Raman peaks located at approximately 140 cm^−1^ (Eg), 398 cm^−1^ (B1g), 525 cm^−1^ (Eg), and 640 cm^−1^ (Eg) corresponded to anatase vibrational modes and were consistent with literature data [44,45].

The anatase phase fraction increased with the heating/cooling rate, reaching 56%, 61%, and 62% for heating/cooling rates of 6 °C/min, 10 °C/min, and 20 °C/min, respectively. This trend indicated that higher thermal rates promoted crystallization and may have influenced the defect structure, potentially affecting electrochemical behavior.

Annealing significantly enhanced structural ordering, as evidenced by the increased intensity and definition of Raman bands. This observation was consistent with previous studies demonstrating that thermal treatment induced the transformation of amorphous TiO_2_ into crystalline phases, predominantly anatase within the temperature range of 300–600 °C [45,46]. An increase in heating/cooling rate, particularly up to 20 °C/min, appears to correlate with a rise in Raman intensity, indicative of enhanced crystallinity. Previous studies suggest that rapid heating/cooling rates may favor the anatase phase by providing sufficient energy for atomic rearrangements while minimizing grain growth, thereby optimizing structural ordering within the material [33].

The positive relationship between heating/cooling rate and Raman intensity may also reflect improved connectivity and alignment within the TiO_2_ lattice, as higher heating/cooling rates facilitate more rapid nucleation of anatase crystallites [47,48]. Enhanced Raman active modes under faster thermal conditions suggest that these rates may be ideal for applications where high anatase content and crystallinity are advantageous, such as in photocatalysis, where anatase’s unique electronic and optical properties are critical [49]. Thus, our findings support the potential of controlled heating/cooling rates to tailor the crystalline quality and functional properties of TiO_2_ nanotubes.

Table 2 presents the specific Raman spectral features associated with the anatase peak at 525 cm^−1^ [50]. These results confirmed the correlation between the anatase content in hTNTs annealed at varying heating/cooling rates and the normalized Raman intensity. No relationship was observed between peak width or peak position and variations in the heating and cooling rates.

**Figure 3:**
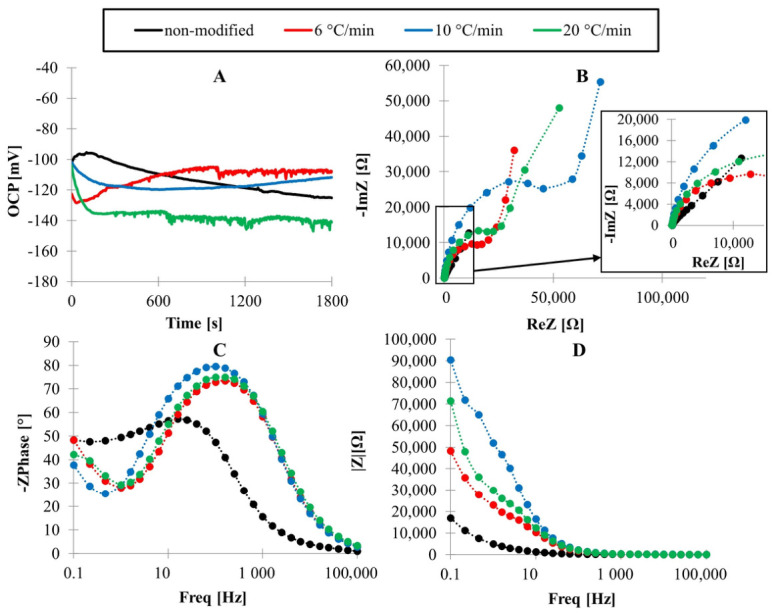
Open-circuit potential curves (**A**), Nyquist plot (**B**), and Bode plots (**C**,**D**) of non-modified and annealed hTNTs treated in air at 550 °C with heating/cooling rates of 6 °C/min, 10 °C/min, and 20 °C/min. Measurements were conducted in Ringer’s solution.

Figure 3A shows the open-circuit potential evolution of hexagonal TiO_2_ nanotubes annealed using different heating/cooling rates, together with the non-annealed sample. All samples exhibited similar initial OCP values of approximately −100 mV, indicating comparable initial electrode/electrolyte interfacial conditions. Non-annealed hTNTs exhibited a gradual decrease in OCP to approximately −125 mV vs. Ag/AgCl over 1800 s, with slower potential stabilization compared with annealed samples. In contrast, hTNTs annealed at 6 °C/min showed an initial increase in OCP to approximately −110 mV within the first 800 s, followed by subsequent stabilization. The hTNTs annealed at 550 °C with heating/cooling rate of 10 °C/min initially decreased to approximately −120 mV, and then gradually shifted toward less negative values. Conversely, hTNTs annealed at 20 °C/min exhibited a rapid decrease in OCP to approximately −135 mV, followed by stabilization near −140 mV. The observed differences in OCP evolution indicate that both annealing and heating/cooling rate significantly influence the electrochemical state of the hTNT/electrolyte interface. The shift of stabilized OCP values toward more negative potentials observed for hTNTs annealed at higher heating/cooling rates was likely associated with thermally induced modifications of the oxide layer structure. Faster thermal rates promoted more extensive crack formation and may have limited defect relaxation during annealing, leading to changes in surface defect distribution and electrolyte accessibility within the nanotubular layer. These structural changes could modify the interfacial charge distribution and electrochemical balance at the hTNT/electrolyte interface, resulting in altered OCP behavior.

The Nyquist plot (Figure 3B) illustrated significant differences in the electrochemical impedance response of hexagonal titanium dioxide nanotubes (hTNTs) subjected to annealing with different heating/cooling rates. Non-annealed hTNTs exhibited the lowest impedance values, characterized by the smallest semi-circle diameter in the high-frequency region and a rapid increase in both the real (ReZ) and imaginary (-ImZ) components, reaching approximately 15,000 Ω in ReZ. Annealed hTNTs exhibited substantially higher impedance responses compared with non-annealed samples. hTNTs annealed with heating/cooling rates of 6 °C/min demonstrated a moderate increase in impedance, with a maximum −ImZ value of approximately 30,000 Ω. Increasing the heating/cooling rate to 10 °C/min resulted in a broader impedance response and a higher maximum −ImZ value approaching 40,000 Ω. The sample annealed with 20 °C/min exhibited the highest impedance response over the entire investigated frequency range, indicating significantly increased resistance to interfacial charge transfer processes. The semi-circles observed in the high-frequency region are associated with the charge transfer resistance (Rct) at the hTNT/electrolyte interface. The smallest semi-circle obtained for the non-annealed hTNTs indicated the lowest Rct values and the most favorable charge transfer kinetics. In contrast, the progressive enlargement of the semi-circle diameter with increasing heating/cooling rate indicated increased charge transfer resistance after thermal treatment. In the low-frequency region, all samples exhibited a linear region with an approximate slope of 45°, characteristic of Warburg impedance associated with diffusion-controlled processes. The increasingly pronounced Warburg behavior observed at higher heating/cooling rates indicated increasing limitations in ion diffusion within the nanotubular oxide layer. These effects were likely associated with structural or morphological changes, such as reduced pore accessibility or increased defect density, that limit mass transport.

The Bode plots shown in Figure 3C,D illustrate the phase angle (θ) and impedance modulus (|Z|) recorded for hexagonal titanium dioxide nanotubes thermally modified at 550 °C in air for 2 h using heating/cooling rates of 6 °C/min, 10 °C/min, and 20 °C/min, alongside an unannealed control. All samples exhibited characteristic phase angle maxima within the investigated frequency range, associated with charge transfer and capacitive processes at the hTNT/electrolyte interface. The non-annealed hTNTs exhibited the highest phase angle values at lower frequencies compared with thermally treated samples. Among the thermally treated samples, hTNTs treated with 6 °C/min exhibited slightly higher phase angle values than hTNTs annealed with 10 °C/min and 20 °C/min. Additionally, decreasing heating/cooling rate shifted the phase angle toward lower frequencies indicating changes in interfacial electrochemical behavior after thermal treatment. Significant differences were also observed in the impedance modulus spectra. Non-annealed hTNTs showed the lowest impedance modulus values over the investigated frequency range, whereas the sample annealed with heating/cooling rate of 10 °C/min demonstrated the highest impedance modulus. The samples annealed with 6 °C/min and 10 °C/min exhibited intermediate impedance responses.

The observed differences in phase angle and impedance modulus indicated that thermal heating/cooling rates significantly affected the electrochemical response of hTNTs. Thermal treatment modified the phase composition and structural organization of the oxide layer, which influenced charge transfer resistance and diffusion-related processes within the nanotubular structure. The lower impedance response observed for non-annealed hTNTs was associated with the predominantly amorphous character of the oxide layer, whereas annealed samples exhibited increased impedance following crystallization and structural reorganization induced during thermal treatment.

**Figure 4:**
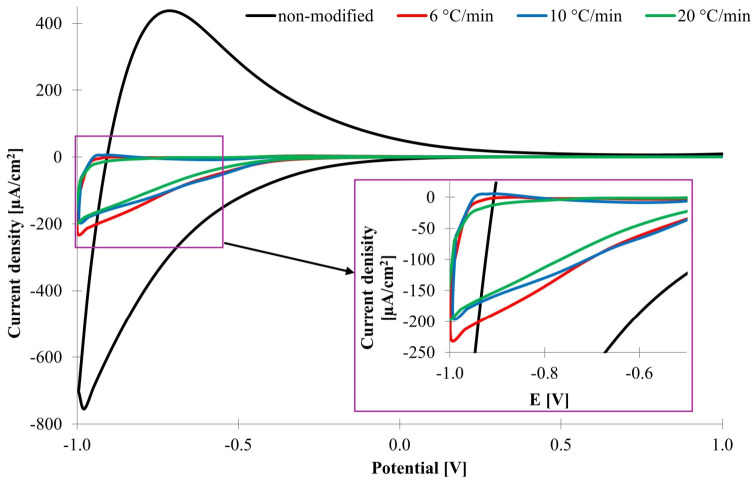
Cyclic voltammetry plots of hTNTs before and after annealing in air at 550 °C for 2 h with heating/cooling rates of 6 °C/min, 10 °C/min, and 20 °C/min. Measurements were conducted in Ringer’s solution.

The cyclic voltammetry curves in Figure 4 show the electrochemical response of hTNTs before and after thermal treatment using different heating/cooling rates. The non-annealed hTNTs exhibited the highest anodic and cathodic current densities among all investigated samples. This behavior resulted from both enhanced charge transfer and increased charge accumulation in the amorphous oxide layer. The high density of structural defects and localized states in amorphous TiO_2_ can facilitate charge storage, contributing to the overall current response. Additionally, the non-annealed samples also exhibited a pronounced asymmetry of the CV profile, which may be attributed to structural disorder and oxygen-related defects present in the amorphous TiO_2_ layer. Such defects may act as charge trapping centers, leading to capacitive charge accumulation and influencing the shape and asymmetry of the voltammograms. Therefore, the observed electrochemical response is likely governed by a combination of faradaic charge transfer and non-faradaic charge storage processes.

Thermal treatment resulted in a significant decrease in both anodic and cathodic current densities for all annealed hTNTs. This behavior was observed after thermal treatment, which led to increased crystallinity of TiO_2_, as indicated by Raman spectroscopy (Figure 2). The CV curves of all thermally treated samples did not exhibit distinct redox peaks, indicating that the electrochemical response was dominated by surface-controlled Ti^4+^/Ti^3+^ processes rather than diffusion-limited electrochemical reactions. Among the annealed samples, hTNTs treated with a heating/cooling rate of 6 °C/min exhibited the most negative cathodic currents, indicating comparatively higher reduction activity. This behavior may be related to a greater retention of structural defects, which can act as active sites for charge transfer. In contrast, increasing the heating/cooling rate to 10 °C/min and 20 °C/min resulted in a gradual reduction in cathodic current density, suggesting changes in crystallinity and defect structure.

**Figure 5:**
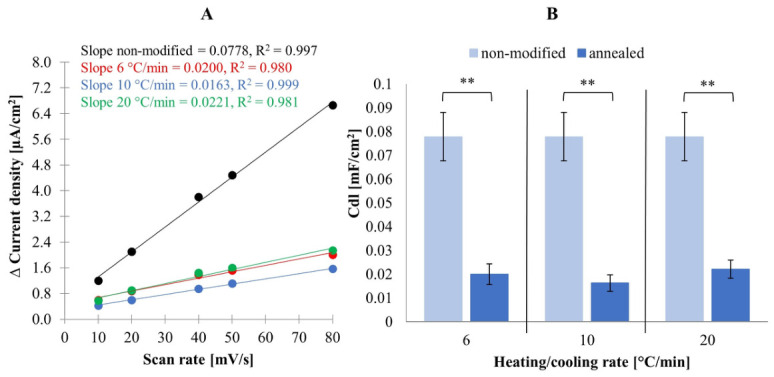
Plot of current density at 200 mV versus scan rates (**A**) and double-layer capacitance (Cdl) (**B**) of hTNTs before and after annealing in air at 550 °C for 2 h with heating/cooling rates of 6 °C/min, 10 °C/min, and 20 °C/min obtained by cyclic voltammetry. Measurements were conducted in Ringer’s solution. ** *p* < 0.01 versus un-modified hTNT sample (Welch’s *t*-test).

Cyclic voltammetry measurements conducted in the non-faradaic potential range of 100–300 mV at scan rates of 10, 20, 40, 50, and 80 mV/s revealed a linear increase in current density with increasing scan rate for all investigated samples (Figure 5A). These correlations confirm the predominantly capacitive nature of the electrochemical response. The highest double-layer capacitance (Cdl) was observed for the non-modified hTNTs sample (Figure 5B). Annealing resulted in a reduction in Cdl regardless of the applied heating/cooling rate (6, 10, and 20 °C/min). No distinct relationship between the heating/cooling rate and Cdl was observed. The decrease in Cdl after annealing suggests a reduction in the electrochemically active surface area and/or changes in the surface properties of the nanotubular layer. However, the obtained results do not indicate a clear influence of the heating/cooling rate on the capacitive behavior of the investigated samples.

### 3.2. Influence of Annealing Temperature on Phase Composition and Electrochemical Properties of hTNTs

**Figure 6:**
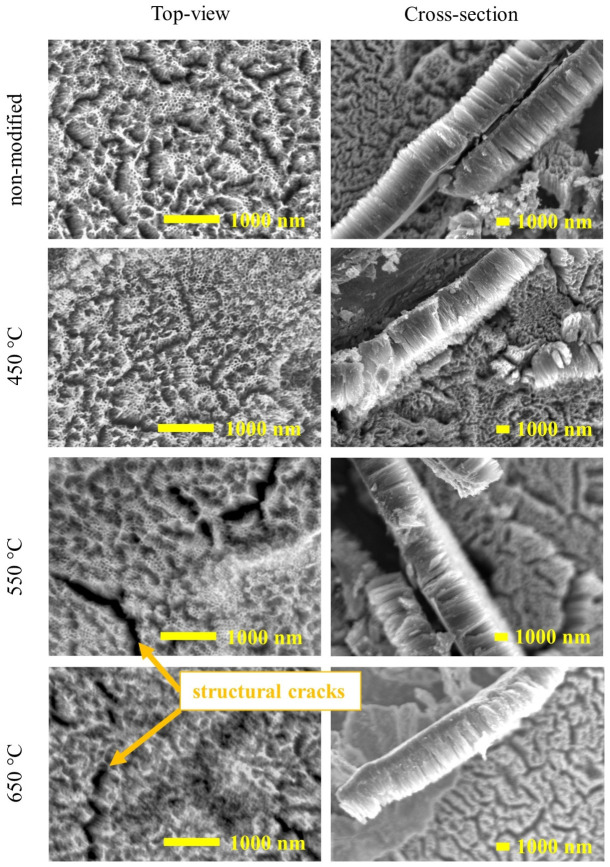
SEM images of hTNTs annealed in air for 2 h at 450 °C, 550 °C, and 650 °C using a heating/cooling rate of 10 °C/min.

As presented in Figure 6, SEM analysis confirmed that hTNTs preserved their hexagonal morphology after annealing at 450 °C, 550 °C, and 650 °C in air [51]. Simultaneously, increasing annealing temperature promoted the formation of cracks within the oxide layer, indicating enhanced thermally induced stress development during crystallization and phase transformation processes. The morphological parameters and elemental composition of hTNTs annealed at different temperatures are summarized in Table 3. Annealing temperature did not significantly affect nanotube diagonal and height, with values remaining close to 43 ± 4 nm and 3755 ± 319 nm, respectively. Similar preservation of nanotubular morphology after annealing in air has also been reported for conventional circular TiO_2_ nanotubes [49].

EDS analysis confirmed the removal of fluorine ions from the surface of the hTNTs after annealing, in agreement with the results discussed previously. The highest oxygen content was observed in the sample annealed at 550 °C. This sample had a Ti/O ratio closest to stoichiometric TiO_2_, which may indicate reduced oxygen deficiency within the oxide layer. Minor variations in the Ti/O ratio were observed among samples annealed at different temperatures, suggesting no substantial change in titanium dioxide stoichiometry. Nevertheless, increasing annealing temperature influenced crack morphology and may have affected the distribution of oxygen-related defect states within the oxide layer.

**Figure 7:**
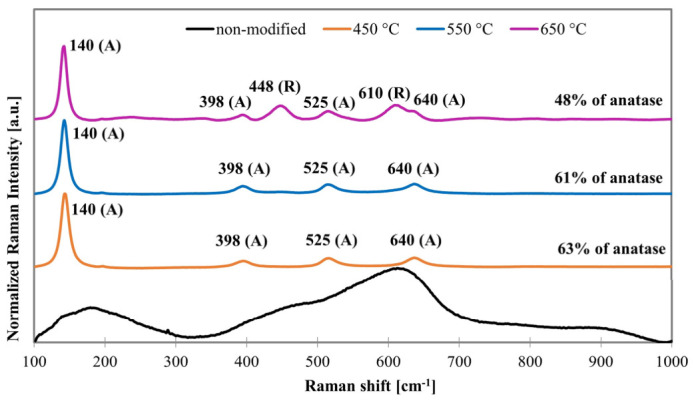
Raman spectra of hTNTs before and after annealing in air for 2 h at different temperatures (450–650 °C) using a heating/cooling rate of 10 °C/min.

The Raman spectra presented in Figure 7 demonstrate the evolution of the crystalline structure of TiO_2_ nanotubes as a function of annealing temperature. Characteristic Raman peaks corresponding to the anatase phase (A) are clearly observed at approximately 140 cm^−1^ (Eg), 398 cm^−1^ (B1g), 525 cm^−1^ (Eg), and 640 cm^−1^ (Eg), which are associated with the vibrational modes of anatase TiO_2_ and consistent with values reported in the literature [52,53]. These peaks were present after annealing at 450 °C, 550 °C and 650 °C, confirming the formation of the anatase phase as a result of thermal treatment.

The anatase content decreases with increasing annealing temperature, reaching 63%, 61%, and 48% at temperatures of 450 °C, 550 °C, and 650 °C, respectively. At 650 °C, additional Raman peaks appeared at approximately 448 cm^−1^ and 610 cm^−1^, which are characteristic of the rutile phase (R) of TiO_2_. The emergence of these peaks indicates the onset of the anatase-to-rutile phase transformation, which is commonly reported for TNTs subjected to elevated temperatures [26,46]. Despite the presence of rutile-related modes, the dominant peaks in the spectrum still correspond to anatase, suggesting that anatase remains the prevailing crystalline phase under these conditions. In contrast, the non-modified sample showed broad and weak spectral features, characteristic of predominantly amorphous TiO_2_.

Table 4 presents the specific Raman spectral features associated with the anatase peak at 525 cm^−1^. No changes in the position of the anatase peak were observed with increasing annealing temperature of hTNTs. However, a decrease in both the intensity and width of the anatase peak was observed with increasing annealing temperature. These results may be related to changes in the unit cell associated with increased crystallinity and anatase-to-rutile transformation [46].

**Figure 8:**
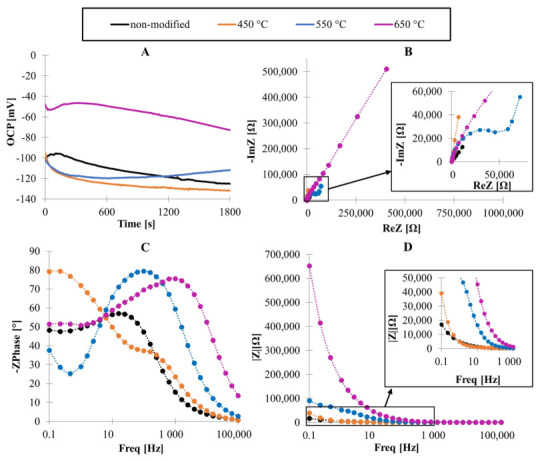
Open-circuit potential curves (**A**), Nyquist plots (**B**), and Bode plots (**C**,**D**) of non-modified and annealed hTNT samples at heating/cooling rates of 10 °C/min for 2 h in air with different temperatures of 450 °C, 550 °C and 650 °C. Measurements were performed in Ringer’s solution.

Figure 8A presents the evolution of the open-circuit potential (OCP) hexagonal TiO_2_ nanotubes annealed at different temperatures (450 °C, 550 °C, and 650 °C), together with a non-annealed reference sample. Non-annealed hTNTs exhibited an initial OCP value close to −95 mV, which gradually decreased to approximately −125 mV during the 1800 s measurement period, indicating progressive stabilization of the hTNT/electrolyte interface. A similar decreasing trend was observed for the sample annealed at 450 °C, although the stabilized potential reached slightly more negative values, near −132 mV. hTNTs annealed at 550 °C exhibited a rapid initial decrease in OCP to approximately −120 mV, followed by a gradual shift toward less negative values, stabilizing near −112 mV. In contrast, the sample annealed at 650 °C exhibited distinctly different behavior, characterized by an initial potential near −73 mV and a relatively stable profile over most of the measurement time, followed by a slight increase at longer times. The observed differences in OCP evolution indicated that annealing temperature substantially affected the interfacial electrochemical behavior of hTNTs. Increasing annealing temperature shifted the stabilized OCP toward more positive values, which may have been associated with modifications in surface chemistry and electronic structure associated with increased crystallinity and possible phase transformations.

The Nyquist plot shown in Figure 8B illustrates the impedance characteristics of the hTNTs annealed at different temperatures. The sample annealed at 450 °C showed a moderate impedance response with a relatively small semi-circle in the low-frequency region, suggesting the presence of charge transfer processes combined with diffusion effects. The sample treated at 550 °C exhibited a larger semi-circle, indicating increased charge transfer resistance and altered electrochemical kinetics. The highly resistive electrochemical response is observed for the sample annealed at 650 °C, where the Nyquist plot displays a nearly linear increase in both the real and imaginary parts of impedance, reaching the highest impedance modulus values among all samples. This behavior indicates a pronounced increase in charge transfer resistance and a suppression of well-defined capacitive and diffusion-related contributions. Such changes suggest a transition toward a transport-limited regime, which can be associated with rutile formation, reduced electronic conductivity, and thermal densification of the nanotubular architecture, leading to increased resistance for both electron and ion transport. The lack of a distinct Warburg impedance contribution further suggests a strongly limited ionic diffusion within the nanotubular layer at elevated annealing temperatures. Differences were also observed in the low-frequency region of the spectra. A diffusion-related response characteristic of Warburg impedance was most evident for hTNTs annealed at 550 °C, indicating the contribution of ion diffusion processes within the nanotubular oxide layer. In contrast, the sample annealed at 450 °C did not exhibit a pronounced Warburg-type response, which can be attributed to relatively fast ion transport and low charge transfer resistance. For hTNTs annealed at 650 °C, the impedance response was dominated by significantly increased charge transfer resistance, which reduced the contribution of diffusion-related processes within the investigated frequency range.

The Bode plots presented in Figure 8C,D show the influence of annealing temperature on the phase angle and impedance modulus of hTNTs. All samples exhibited characteristic phase angle maxima within specific frequency ranges, associated with charge transfer and capacitive processes occurring at the hTNT/electrolyte interface. The non-annealed hTNTs exhibited a moderate phase angle maximum at intermediate frequencies, indicating relatively low charge transfer dynamics. In contrast, thermally treated samples exhibited more pronounced phase angle maxima accompanied by shifts in their frequency position. The sample annealed at 450 °C shows a broad phase angle maximum shifted toward lower frequencies, suggesting enhanced capacitive behavior associated with the formation of crystalline anatase structures. hTNTs annealed at 550 °C exhibited the highest phase angle value among the thermally treated samples, indicating improved electrochemical activity and charge storage capability. Meanwhile, the sample annealed at 650 °C demonstrated a broader phase response and shifted toward higher frequencies. The shift of the phase angle maximum toward higher frequencies indicates a reduction in the characteristic time constants of the system, consistent with hindered charge storage and interfacial charge transfer kinetics.

The non-annealed sample exhibited relatively low impedance modulus values, indicating lower resistance to charge transfer at the electrode–electrolyte interface. In comparison, thermally treated samples demonstrated a pronounced increase in impedance modulus, particularly with increasing annealing temperature. The highest impedance modulus was observed for the sample annealed at 650 °C. An increase in impedance modulus means lower conductivity and potentially greater resistance to charge transfer or diffusion processes.

**Figure 9:**
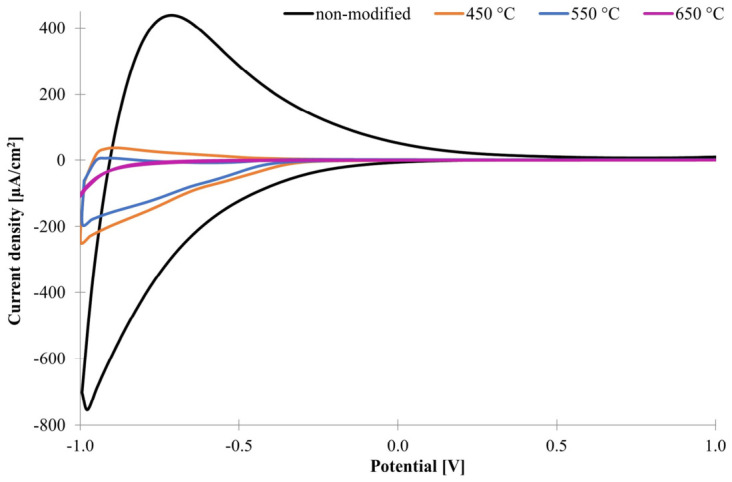
Cyclic voltammograms of hTNTs before and after annealing in air for 2 h with heating/cooling rate of 10 °C/min at different temperatures of 450 °C, 550 °C and 650 °C. Measurements were performed in Ringer’s solution.

The cyclic voltammetry curves presented in Figure 9 illustrate the electrochemical response of hexagonal titanium dioxide nanotubes, including a non-modified sample and samples thermally treated at different annealing temperatures (450 °C, 550 °C, and 650 °C). Non-annealed hTNTs exhibited the highest anodic and cathodic current densities among all investigated samples, indicating charge transfer activity. The pronounced asymmetry in the CV profile was observed, which may be associated with a high amount of oxygen vacancies and the presence of an amorphous TiO_2_ phase. The decrease in both anodic and cathodic current densities was observed for all annealed samples compared to non-modified hTNTs. This behavior was associated with increased crystallinity indicated by annealing. The CV curves of the thermally treated samples exhibited profiles without redox peaks, suggesting that the electrochemical response is dominated by surface-controlled Ti^4+^/Ti^3+^ processes. Among the annealed samples, the specimen treated at 450 °C exhibited the highest current densities, indicating relatively higher electrochemical activity compared to the samples annealed at higher temperatures. Increasing the annealing temperature to 550 °C resulted in a further reduction in current response, while the sample treated at 650 °C showed the lowest current densities and the most flattened CV profile.

**Figure 10:**
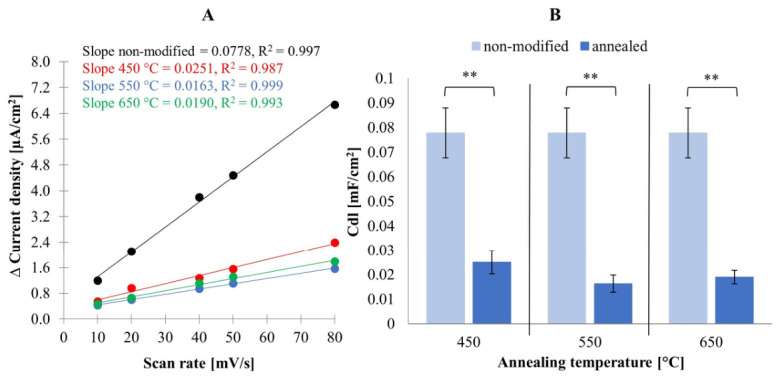
Plot of current density at 200 mV versus scan rates (**A**) and double-layer capacitance (Cdl) (**B**) of hTNTs before and after annealing in air for 2 h with heating/cooling rate of 10 °C/min at different temperatures of 450 °C, 550 °C and 650 °C obtained by cyclic voltammetry. Measurements were conducted in Ringer’s solution. ** *p* < 0.01 versus un-modified hTNT sample (Welch’s *t*-test).

Cyclic voltammetry measurements performed in the non-faradaic potential range of 100–300 mV at scan rates of 10, 20, 40, 50, and 80 mV s^−1^ revealed a linear increase in current density with increasing scan rate for all investigated samples (Figure 10A). The non-modified hTNTs exhibited the highest double-layer capacitance (Figure 10B). Thermal treatment resulted in a decrease in Cdl compared to the non-annealed sample, indicating that annealing affected the electrochemical characteristics of the nanotubular surface. However, no clear relationship between the annealing temperature and the obtained Cdl values was observed within the investigated temperature range.

### 3.3. Summary

Table 5 summarizes the most important results obtained for the hexagonal titanium dioxide nanotubes annealed in air at 450 °C, 550 °C and 650 °C for 2 h, with varying heating/cooling rates (6 °C/min, 10 °C/min and 20 °C/min). Variations in annealing temperature and heating/cooling rate did not significantly affect the diagonal or height of the hexagonal TiO_2_ nanotubes. In contrast, thermal treatment strongly affected the phase composition and electrochemical behavior of hTNTs. Annealed hTNTs exhibited formation of the crystalline anatase phase, the content of which increased with the heating/cooling rate and decreased at 650 °C due to partial anatase-to-rutile transformation. Annealing and heating/cooling rates significantly impact OCP, double-layer capacitance and electrochemical stability, with faster heating/cooling rates and higher temperatures enhancing initial electrochemical reactivity. Non-annealed hTNTs exhibited the lowest impedance modulus, whereas the sample annealed at 650 °C using heating/cooling rate of 10 °C/min demonstrated highest impedance response among all investigated samples. The obtained results demonstrated that thermal treatment kinetics significantly affected phase evolution, charge transfer conditions, and diffusion-related processes within the hexagonal nanotubular oxide layer.

## 4. Conclusions

Hexagonal TiO_2_ nanotubes formed by sonoelectrochemical anodization were thermally modified in air at different temperatures and with different heating/cooling rates in order to investigate the relationship between thermal treatment kinetics, phase evolution, and electrochemical behavior. Microscopic and structural analyses of hTNTs confirmed no substantial impact of the hexagonal geometry after thermal modification, accompanied by controlled anatase transformation and fluorine removal. The content of anatase phase increased with the heating/cooling rate in order to investigate the relationship between thermal treatment kinetics, phase evolution, and electrochemical behavior. Annealing and the applied heating/cooling rate markedly influenced the open-circuit potential and electrochemical stability of hTNTs, with more rapid heating/cooling and higher temperatures promoting higher initial electrochemical reactivity. The non-annealed hTNTs exhibited the lowest impedance modulus, whereas the sample cooled at 10 °C/min and 650 °C showed the highest impedance. The transformation of amorphous TiO_2_ into a more ordered crystalline structure after heat treatment of hTNT results in a significant reduction in both anodic and cathodic current.

Importantly, this work constitutes the first systematic investigation of the influence of annealing heating/cooling rates on hTNTs. The identified relationships between thermal treatment parameters, phase transformations, and functional performance establish a solid basis for the rational development of hTNT-based coatings with controlled properties, suitable for advanced surface engineering.

## Figures and Tables

**Table 1:** Morphological parameters and EDS elemental composition of as-fabricated and annealed (air, 550 °C, 2 h, heating/cooling rate: 6 °C/min, 10 °C/min and 20 °C/min) hexagonal TiO_2_ nanotubes.

	Morphology		EDS Analysis		
Heating/Cooling Rate	Diagonal [nm]	Height [nm]	Ti [wt.%]	O [wt.%]	F [wt.%]
non-modified	43 ± 4	3755 ± 319	62.82 ± 1.12	29.46 ± 1.10	7.73 ± 0.29
6 °C/min	41 ± 1	3356 ± 360	67.91 ± 0.28	32.09 ± 0.45	-
10 °C/min	45 ± 3	3420 ± 204	65.34 ± 0.48	34.66 ± 0.52	-
20 °C/min	41 ± 4	3502 ± 389	67.25 ± 1.05	32.75 ± 0.54	-

**Table 2:** Specific Raman spectral features of as-fabricated and annealed (air, 550 °C, 2 h, heating/cooling rate: 6 °C/min, 10 °C/min and 20 °C/min) hexagonal TiO_2_ nanotubes with emphasis on the anatase peak at ~525 cm^−1^.

Heating/Cooling Rate	Raman Shift [cm^−1^]	Normalized Raman Intensity [a.u.]	with [cm^−1^]
non-modified	-	-	-
6 °C/min	525.23 ± 0.25	0.1066 ± 0.0005	22.69 ± 0.58
10 °C/min	525.23 ± 0.27	0.1264 ± 0.0006	23.54 ± 0.46
20 °C/min	525.21 ± 0.31	0.1299 ± 0.0008	22.73 ± 0.65

**Table 3:** Morphological parameters and EDS elemental composition of as-fabricated and annealed hTNTs treated in air for 2 h at 450 °C, 550 °C, and 650 °C using a heating/cooling rate of 10 °C/min.

	Morphology		EDS Analysis		
Annealing Temperature	Diagonal [nm]	Height [nm]	Ti [wt.%]	O [wt.%]	F [wt.%]
non-modified	43 ± 4	3755 ± 319	62.82 ± 1.12	29.46 ± 1.10	7.73 ± 0.29
450 °C	44 ± 3	3315 ± 115	69.39 ± 0.38	30.61 ± 0.25	-
550 °C	45 ± 3	3420 ± 204	65.34 ± 1.01	34.66 ± 0.61	-
650 °C	47 ± 2	3720 ± 272	67.91 ± 0.28	32.09 ± 0.42	-

**Table 4:** Specific Raman spectral features of as-fabricated and annealed hTNTs treated in air for 2 h at 450 °C, 550 °C, and 650 °C using a heating/cooling rate of 10 °C/min with emphasis on the anatase peak at ~525 cm^−1^.

Heating/Cooling Rate	Raman Shift [cm^−1^]	Normalized Raman Intensity [a.u.]	with [cm^−1^]
non-modified	-	-	-
450 °C	525.63 ± 0.20	0.1333 ± 0.0011	24.52 ± 0.39
550 °C	525.23 ± 0.27	0.1264 ± 0.0006	23.54 ± 0.46
650 °C	525.33 ± 0.29	0.0858 ± 0.0015	21.48 ± 0.48

**Table 5:** Summary of the results obtained for non-annealed and annealed hTNTs treated in the air for 2 h at 450 °C, 550 °C and 650 °C with heating/cooling rates of 6 °C/min, 10 °C/min, and 20 °C/min.

Annealing Temperature	Heating/Cooling Rate	Diagonal [nm]	Height [nm]	Anatase Content [%]	OCP [mV]	|Z| [Ω]
non-modified	Non	43 ± 4	3755 ± 319	-	−125 ± 19	17,004 ± 2900
450 °C	10 °C/min	44 ± 3	3315 ± 115	63	−132 ± 20	38,927 ± 3428
550 °C	6 °C/min	41 ± 1	3356 ± 360	56	−108 ± 17	48,362 ± 3265
	10 °C/min	45 ± 3	3420 ± 204	61	−112 ± 20	90,494 ± 4204
	20 °C/min	41 ± 4	3502 ± 389	62	−141 ± 18	71,365 ± 3587
650 °C	10 °C/min	47 ± 2	3720 ± 272	48	−73 ± 16	651,797 ± 2879

## Data Availability

The data supporting the conclusions of this article will be made available by the corresponding author on request.
